# Morphotypes and genetic diversity of Dendrobaena schmidti (Lumbricidae, Annelida)

**DOI:** 10.18699/VJ20.594

**Published:** 2020-02

**Authors:** S.V. Shekhovtsov, I.B. Rapoport, T.V. Poluboyarova, A.P. Geraskina, E.V. Golovanova, S.E. Peltek

**Affiliations:** Institute of Cytology and Genetics of Suberian Branch of the Russian Academy of Sciences, Novosibirsk, Russia Institute of Biological Problems of the North of Far Eastern Branch of the Russian Academy of Sciences, Magadan, Russia Novosibirsk State University, Novosibirsk, Russia; Tembotov Institute of Ecology of Mountain Territories of the Russian Academy of Sciences, Nalchik, Russia; Institute of Cytology and Genetics of Suberian Branch of the Russian Academy of Sciences, Novosibirsk, Russia Institute of Biological Problems of the North of Far Eastern Branch of the Russian Academy of Sciences, Magadan, Russia; Center for Forest Ecology and Productivity of the Russian Academy of Sciences, Moscow, Russia; Omsk State Paedagogical University, Omsk, Russia; Institute of Cytology and Genetics of Suberian Branch of the Russian Academy of Sciences, Novosibirsk, Russia

**Keywords:** earthworms, Dendrobaena schmidti, cox1, ITS2, phylogeny, дождевые черви, Dendrobaena schmidti, cox1, ITS2, филогения.

## Abstract

Dendrobaena schmidti (Michaelsen, 1907) is a polymorphic earthworm species from the Caucasus and adjacent regions. Adult D. schmidti individuals have highly variable body size (from 1.5 to well over 10 cm) and color (from dark purple to total lack of pigmentation), so a lot of subspecies of D. schmidti have been described; however, the existence of most of them is currently under dispute. We studied the genetic diversity of D. schmidti from seven locations from the Western Caucasus using mitochondrial (a fragment of the cytochrome oxidase I gene) and nuclear (internal ribosomal transcribed spacer 2) DNA. For both genes studied, we found that our sample was split into two groups. The first group included somewhat bigger (3–7.5 cm) individuals that were only slightly pigmented or totally unpigmented (when fixed by ethanol). The second group contained small (1.7–3.5 cm) specimens with dark purple pigmentation. In one of the studied locations these two groups were found in sympatry. However, there were no absolute differences either in general appearance (pigmented/unpigmented, small/big) or among diagnostic characters. Although the two groups differed in size (the majority of individuals from the first group were 5–6 cm long, and of the second one, 2–3 cm), the studied samples overlapped to a certain degree. Pigmentation, despite apparent differences, was also unreliable, since it was heavily affected by fixation of the specimens. Thus, based on the obtained data we can conclude that D. schmidti consists of at least two species that have identical states of diagnostic characters, but differ in general appearance.

## Abstract (ru)

Dendrobaena schmidti – полиморфный вид дождевых червей, обитающий на Кавказе и в сопредельных регионах. Особи D. schmidti отличаются большим диапазоном размеров (от 1.5 до 10 см и более) и пигментации (от интенсивной пурпурово-фиолетовой окраски до полного ее отсутствия). В связи с этим исследователями описано множество подвидов D. schmidti, правомерность выделения большинства из которых в настоящее время оспаривается. В настоящей работе нами изучена генетическая изменчивость выборки D. schmidti из семи точек Северо-Западного Кавказа с использованием митохондриальной (фрагмент гена цитохромоксидазы I) и ядерной (внутренний рибосомальный транскрибируемый спейсер 2) ДНК. По обоим маркерам выборка разделилась на две группы. В первую вошли более крупные (3–7.5 см) черви, непигментированные или со слабо выраженной на передней половине тела окраской у зафиксированных в спирте особей. Вторую группу представляли только мелкие (1.7–3.5 см) особи с выраженной пурпурной пигментацией (тоже у фиксированных в спирте образцов). В одной из изученных географических точек обе группы сосуществовали в симпатрии. При этом абсолютных различий между данными видами ни по внешнему виду (пигментированные/непигментированные, мелкие/крупные), ни по диагностическим признакам установить не удалось: хотя выборки особей первой и второй групп различались по размеру (в первой груп- пе большинство особей имело длину 5–6 см, во второй – 2–3 см), они, тем не менее, перекрывались по этому параметру. По пигментации между найденными группами также возможно заметное перекрытие, учитывая, что оценку окраски червей на практике осуществляют посмертно после фиксации, которая влияет на резуль- таты анализа. Таким образом, на основании полученных нами данных можно заключить, что в пределах вида D. schmidti существуют как минимум два вида, сходных по диагностическим признакам, но различающихся внешне.

## Introduction

Earthworms are probably the best studied group among the Annelida. This is due to their important roles in the function and maintenance of soil ecosystems, as well as the fact they are the easiest to spot among segmented worms. Nevertheless, species diversity of local earthworm faunas may differ considerably according to different scientists. This is caused by the paucity of diagnostic morphological characters and considerable intraspecific variation, which is often higher than differences between species. Moreover, the biological species criterion is inapplicable for parthenogenetic earthworms and impracticable for amphimictic ones.

The most important morphological characters in earthworms are the positions of the clitellum and tuberculae pubertatis (Perel, 1979; Vsevolodova-Perel, 1997). However, individuals with identical states of these characters may demonstrate extreme differences in body size and color. This variation is usually attributed to different environmental conditions, but sometimes such individuals can be found in sympatry. Dendrobaena schmidti (Michaelsen, 1907) is an example of such polymorphism. This species is an endemic of the Caucasus. It is often a dominant species in many habitats. While describing this species, Michaelsen (1907) noted the existence of purple and unpigmented forms, and described them as D. schmidti forma surbiensis and D. schmidti forma montana. Body size is also known to vary widely in this species, from 35 to 160 mm, with different forms often found together. In 1966, unpigmented specimens with the clitellum shifted by one segment towards the anterior end were recognized as the parthenogenetic subspecies D. schmidti tellermanica (Perel, 1966); later on, it was isolated into a separate species, D. tellermanica (Vsevolodova-Perel, 2003).

Kvavadze (1985) divided D. schmidti into eight subspecies: the universally acknowledged nominative D. schmidti schmidti and the unpigmented D. schmidti tellermanica; two subspecies initially described by Michaelsen, D. schmidti surbiensis and D. schmidti montana; and four new ones, D. schmidti colchica, D. schmidti marinae, D. schmidti malevichi, and D. schmidti jaloniensis. This splitting was based on body color, the position of tuberculae pubertatis, of spermathecal pores relative to the d setae, and, later on, the form of locomotive and genital setae visualized by scanning electron microscopy (Kvavadze, 1985; Kvavadze et al., 2007). The validity of these subspecies was disputed, because the differences in diagnoses are minor, except for the color and the number of seminal receptacles (Vsevolodova-Perel, 2003). Another taxon under dispute is Dendrobaena baksanensis Pizl 1984 that was initially described from the Baksan gorge but subsequent researchers failed to detect it in that location.

Rapoport (2009) divided D. schmidti into three morphs: epigeic, endogeic, and intermediate ones. Differences in lifestyle result in distinct size, color, body form, and the rate of response to stimulus. Moreover, there are certain differences in the position of the setae ab on papillae.

Thus we can conclude that currently there is no conventional way to split D. schmidti sensu lato into smaller taxa (except for D. tellermanica), because their delimitation is impeded by the paucity of clear morphological differences and by possible geographic diversity: results of different authors depend on the studied population and the methods used. Perel (1982) suggested that differences between the forms were caused by different ploidy. However, according to Bakhtadze et al. (2003, 2005, 2008), all subspecies of D. schmidti are diploids (2n = 36), while D. tellermanica is tetraploid (4n = 72).

DNA analysis has long become a vital part of systematics. It can be applied universally and is better at reflecting phylogeny than methods of cytogenetics, chemosystematics, and electron microscopy. DNA analysis is especially useful for earthworms, which were shown to possess very high cryptic genetic diversity (King et al., 2008; Pérez- Losada et al., 2009; Shekhovtsov et al., 2016b, 2019). In this work we studied the genetic diversity of a sample of D. schmidti from several populations from the Western Caucasus that demonstrated pronounced variation in size and color.

## Materials and methods

**Fig. 1.**
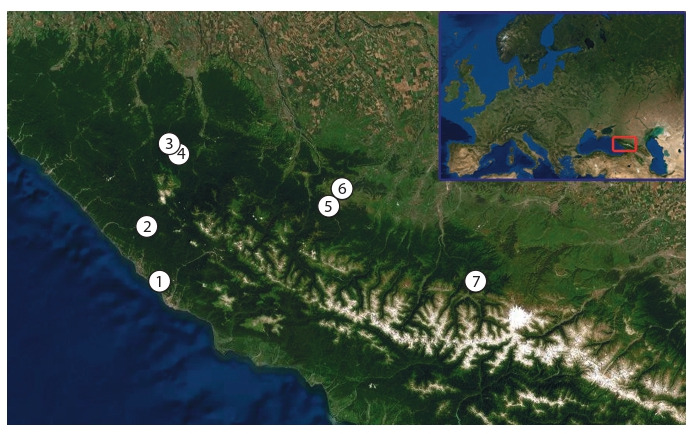
Sampling locations. Location numbers correspond to the numbers in the Table.

D. schmidti individuals were collected in seven locations from the Western Caucasus (Fig. 1, see the Table). Morphological identification was performed according to the key of Vsevolodova-Perel (1997). A piece of body wall (10–50 μg) was excised on the rear body end so that the remaining body was still amenable to morphological analysis and one could count the number of segments and measure body length. Genomic DNA was extracted using silica columns (BioSilica, Russia) according to the manufacturer’s instructions.

**Table 1.**
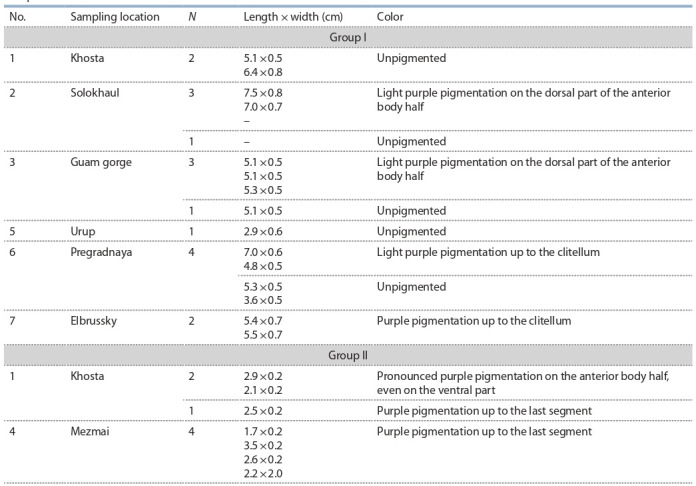
Sampled D. schmidti individuals Note: Sampling location numbers correspond to those in Fig. 1. N, number of individuals. A dash (–) indicates that the individual was damaged so its length could not be determined.

A fragment of the mitochondrial cox1 gene was amplified using universal primers HCO2198 (5ʹ-TAAAC-TTCAG-GGTGA-CCAAA-AAATC-A-3ʹ) and LCO1490m (5ʹ-TACTC-AACAA-ATCAC-AAAGA-TATTG-G-3ʹ) (Folmer et al., 1994; Shekhovtsov et al., 2013). The amplification mix contained 1.5 mM MgCl2, 65 mM Tris-HCl (pH 8.8), 16 mM (NH4)2SO4, 0.05 % Tween-20, 0.2 mM of each deoxynucleotide triphosphate, 0.3 mM of each primer, and 1 U of recombinant TaqSE polymerase (SibEnzyme, Novosibirsk).

A fragment of the ribosomal DNA containing the complete sequence of the internal transcribed spacer 2 (ITS2), as well as partial sequences of the flanking 5.8S and 28S rRNA genes were amplified using universal primers E28S-2 (5ʹ-CC(G/T)CTTCACT- CGCCG-TTA-3ʹ) and E58SF1 (5ʹ-ATCAC-TGGGT-TCGTGCGT- 3ʹ) (Shekhovtsov et al., 2016a). The amplification mixture was similar to that used for cox1, except for the addition of 5 % DMSO needed to disrupt stable secondary structures formed by this DNA fragment.

DNA sequences were determined by Sanger sequencing using BigDye 3.1 kit (Applied Biosystems, USA). Capillary electrophoresis was performed in the SB RAS Genomics Core Facility. Sequences were processed and edited in Chromas (Technelysium Pty Ltd). The final edited sequences were deposited in GenBank (https://www.ncbi.nlm.nih.gov/genbank) under accession numbers MN340181–MN340200, MN340202– MN340205 (cox1) and MN340207–MN340230 (ITS2). Multiple alignments were performed in Clustal Omega (https://www.ebi.ac.uk/Tools/msa/clustalo/). Phylogenetic trees were constructed using Bayesian analysis in MrBayes v.3.2.6 (Ronquist, Huelsenbeck, 2012). The following sequences of various Dendrobaena species from GenBank were used to construct phylogenetic trees; for cox1: D. octaedra (MH755678), D. attemsi (KJ772502), D. veneta (FJ214233), D. karacadagi (MH476311), D. semitica (MH476309), D. pavliceki (MH476308), D. pantaleonis (MH476307), D. orientalis (MH476306), D. hrabei (MH476305); for ITS2: D. octaedra (KX651399), D. byblica (KX651415), D. attemsi (KX651397), D. platyura (KT823916), D. pentheri (KT823915), D. ganglbaueri (KT823909), D. alpina (KX651396, MH469554), D. pantaleonis (MH469555), D. orientalis (MH469553), D. hortensis (MH469549), D. semitica (MH469552), D. karacadagi (MH469547), D. veneta (MH469546). In addition, we used Eisenia fetida (cox1: JX531618; ITS2: JX531571) as an outgroup. MrModeltest v.2 (Nylander, 2004) chose the GTR+I+G substitution model for both sequences used. A total of 10 000 000 replicates of Bayesian analysis was performed; the initial 25 % were discarded as burn-in. The average deviation of split frequencies after analysis was less than 0.01. Nodes with posterior probabilities less than 0.5 were depicted as polytomies.

Values of the Student’s and Welch’s tests were calculated using MathPortal (www.mathportal.org).

## Results

**Fig. 2.**
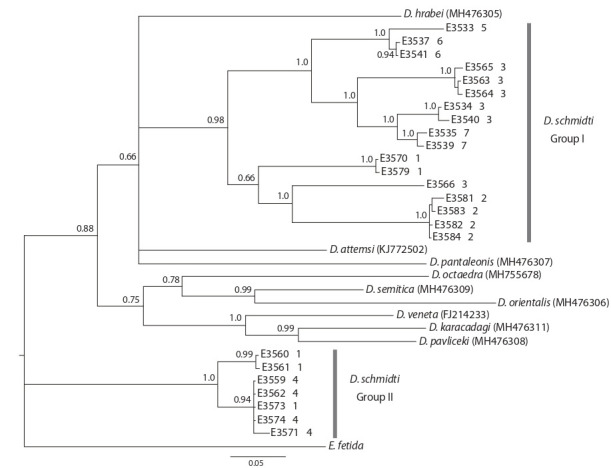
Phylogenetic tree constructed based on cox1 using Bayesian analysis. Posterior probabilities exceeding 0.5 are shown near the nodes; numbers near specimen identifiers denote population numbers given in the Table and Fig. 1.

We obtained cox1 and ITS2 sequences for 24 adult D. schmidti individuals with pronounced diagnostic characters. All cox1 sequences had identical length (658 bp). The phylogenetic tree built based on cox1 data (Fig. 2) suggested that all D. schmidti sequences form two clades referred to as Group I and Group II. Both clades were highly supported by Bayesian posterior probabilities. Both clades were split into several smaller clades.

The length of ITS2 varied widely (549–621 bp). We should note that both rRNA genes and intervening transcribed spacers have complex secondary structures that make their amplification problematic. Earthworm internal transcribed spacers cannot be amplified without denaturing agents, e. g., DMSO. However, even with the addition of DMSO spacers of Group II formed a hairpin, which caused a shorter sequence with an internal deletion of about 78 nucleotides. Thus, this region could not be read clearly and had to be discarded from the alignment.

**Fig. 3.**
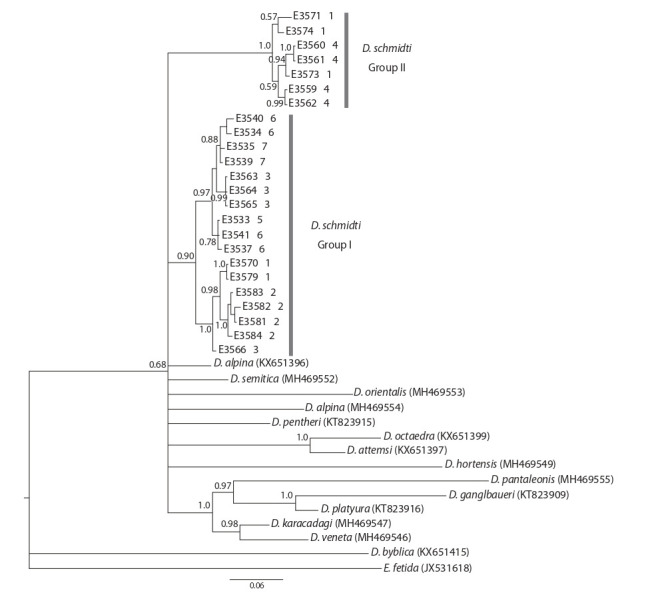
Phylogenetic tree constructed based on ITS2 sequences using Bayesian analysis. Posterior probabilities exceeding 0.5 are shown near the nodes; numbers near specimen identifiers denote population numbers given in the Table and Fig. 1.

According to ITS2 sequences, the studied sample was split into the same groups as for the cox1 tree (Fig. 3). Representatives of both groups were found in sympatry only in location 1 (see Fig. 1).

**Fig. 4.**
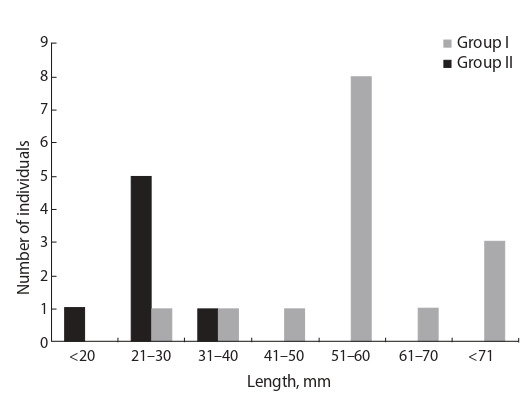
Body length histogram for the studied specimens.

Morphological examination showed that all studied individuals had identical diagnostic character states and could be identified as D. schmidti. However, Groups I and II had certain morphological differences (see the Table). Group I contained unpigmented or weakly pigmented worms; when pigmentation was present, it extended only to the clitellum. Earthworms belonging to Group II were completely or almost completely pigmented, and the color was more intense. There were also certain differences in body size. As seen from Fig. 4, the majority of individuals from Group I were longer than five cm, and those from Group II, shorter than three cm. Length differences between Groups I and II were statistically significant at p < 0.01 according to the Student’s and Welch’s tests.

## Discussion

Systematics and morphological identification of earthworms can be problematic, especially in the cases when intraspecific variation is higher than interspecific variation. Methods of molecular genetics allowed researchers to increase reliability of identification. However, when intraspecific diversity is high, as is the case for D. schmidti, there is again the question as to where to draw the line between potential cryptic species. In our opinion, at the current stage, molecular genetic data can be used as an argument to split a species only in the case when it was proven to be polyphyletic. We can thus conclude that the studied sample from the Western Caucasus contains two groups that can be considered as different species. However, we would abstain from their formal description for the moment. It is worthwhile to note that intraspecific variation within the groups is also high, especially for Group I, with higher distances among its members for the cox1 gene, than, e. g., between D. karacadagi and D. pavliceki (see Fig. 2). Thus, the potential number of species within D. schmidti may be even higher.

The latter viewpoint was also supported by the fact that all specimens from our sample had the state of diagnostic characters typical of D. s. schmidti, and almost all described forms and subspecies had certain deviations from the diagnosis and were found mainly in the southern part of the range, predominantly in Georgia. We can thus hypothesize that a sample collected from a larger territory would help us detect deeper genetic diversity within the D. schmidti complex.

Although we detected no variation in diagnostic characters between Groups I and II, they had pronounced differences in body size and color. These differences were statistically significant, but nevertheless somewhat overlapped. Therefore, discriminating among these two groups based on overall appearance is problematic and can be applied to large samples only.

## Conclusion

Based on the obtained results we can conclude that D. schmidti consists of at least two species that have close diagnoses but vary in general appearance and in DNA sequences. We believe that more cryptic species can be detected by further studies since our work encompassed only a small part of the range of D. schmidti.

## Conflict of interest

The authors declare no conflict of interest.
